# How to Stand

**Published:** 1890-03

**Authors:** W. H. Whitslar

**Affiliations:** Youngstown, Ohio


### How to Stand.

W. H. WHITSLAR, D.D.S., M.D., YOUNGSTOWN, OHIO.

In our enthusiasm over operations at the dental chair we lose all thought of ourselves and think only of the operation we are performing. But in the midst of all this should we neglect our health to accomplish what we, in our mind's eye, conceive to be a skillful and enduring performance? The answer should be positively, *no*! How then can we be faithful to our patients, and yet conserve our well being? One way, amongst the numerous ones, is learning how to stand at the chair in a comfortable manner, and at the same time be able to scrutinize the points to be operated upon. We offer the following suggestions:

It is an easy matter for an operator to fall into the serious habit of assuming positions in standing that are awkward, distorted and cramped, that at once bear ill consequences upon the abdominal viscera as well as the organs of the thoracic cavity.

We naturally assume the position that is easiest, but from point of error, this easy position is oftentimes the wrong one. Many men in operating upon the teeth of the inferior maxilla stand as near in front of the patient as they can, and of course, must use the mirror in looking into most cavities of the teeth. This position, requires an artificial aid. Can it be bettered? We think it can.

A right-handed man naturally stands at the right side of his chair while operating, whereas, the left-handed individual stands on the opposite side.

Now, there are anatomical reasons why both should change, from right to left and left to right, as the case may require when operating, more especially upon the molars and bicuspids. It is the lower jaw only that we are now commenting upon.

Take a jaw bone and notice how the teeth of either side incline; the long axis of each tooth, as a rule, inclines in a line drawn from the lower border of the jaw bone toward the median line, or remains of the anterior suture of the frontal bone. From this, it is not hard to observe that a greater surface view can be obtained of any molar or bicuspid if the operator is standing upon the opposite side of the chair from the side on which the tooth is located.

Often the view is still better if the operator stands a little back of the chair, and sometimes, very often, too, it is still better when he is immediately behind.

With such an advantage in viewing the situation, an erect position, which is most natural, and but a slight inclination of the head is all that is necessary, and hence a greater freedom of the body is obtained, and recourse to mirrors save only for reflected light is often unnecessary. This, we may also add, allows for the patient an easier position of the head and body.

In regard to operating upon teeth of the superior maxilla the ease with which such is accomplished depends largely upon the capability of adjusting the patient's head, and this depends mostly upon the chair we have.

There is no chair too good for our use, and my advice to young men entering the profession is, lay out enough money to get a

*first class* chair that *will* give a wide range of movements, even if it is necessary to let some other seemingly important things go. It is far easier to get the smaller articles afterward than to purchase the ones that are to be life-time companions. Get the *best*, and life and happiness, which means good health, are favorable toward you. But, above all things, *learn how to stand.*

